# Structural Connectivity of Human Inferior Colliculus Subdivisions Using *in vivo* and *post mortem* Diffusion MRI Tractography

**DOI:** 10.3389/fnins.2022.751595

**Published:** 2022-03-22

**Authors:** Kevin R. Sitek, Evan Calabrese, G. Allan Johnson, Satrajit S. Ghosh, Bharath Chandrasekaran

**Affiliations:** ^1^SoundBrain Lab, Brain and Auditory Sciences Research Initiative, Department of Communication and Science Disorders, University of Pittsburgh, Pittsburgh, PA, United States; ^2^Department of Radiology and Biomedical Imaging, University of California, San Francisco, San Francisco, CA, United States; ^3^Center for In Vivo Microscopy, Duke University, Durham, NC, United States; ^4^McGovern Institute for Brain Research, Massachusetts Institute of Technology, Cambridge, MA, United States; ^5^Department of Otolaryngology – Head and Neck Surgery, Harvard Medical School, Boston, MA, United States

**Keywords:** inferior colliculus, diffusion MRI (dMRI), tractography, structural connectivity, human auditory brainstem, subcortical auditory pathway

## Abstract

Inferior colliculus (IC) is an obligatory station along the ascending auditory pathway that also has a high degree of top-down convergence *via* efferent pathways, making it a major computational hub. Animal models have attributed critical roles for the IC in in mediating auditory plasticity, egocentric selection, and noise exclusion. IC contains multiple functionally distinct subdivisions. These include a central nucleus that predominantly receives ascending inputs and external and dorsal nuclei that receive more heterogeneous inputs, including descending and multisensory connections. Subdivisions of human IC have been challenging to identify and quantify using standard brain imaging techniques such as MRI, and the connectivity of each of these subnuclei has not been identified in the human brain. In this study, we estimated the connectivity of human IC subdivisions with diffusion MRI (dMRI) tractography, using both anatomical-based seed analysis as well as unsupervised *k*-means clustering. We demonstrate sensitivity of tractography to overall IC connections in both high resolution *post mortem* and *in vivo* datasets. *k*-Means clustering of the IC streamlines in both the post mortem and *in vivo* datasets generally segregated streamlines based on their terminus beyond IC, such as brainstem, thalamus, or contralateral IC. Using fine-grained anatomical segmentations of the major IC subdivisions, the *post mortem* dataset exhibited unique connectivity patterns from each subdivision, including commissural connections through dorsal IC and lateral lemniscal connections to central and external IC. The subdivisions were less distinct in the context of *in vivo* connectivity, although lateral lemniscal connections were again highest to central and external IC. Overall, the unsupervised and anatomically driven methods provide converging evidence for distinct connectivity profiles for each of the IC subdivisions in both *post mortem* and *in vivo* datasets, suggesting that dMRI tractography with high quality data is sensitive to neural pathways involved in auditory processing as well as top-down control of incoming auditory information.

## Introduction

Inferior colliculus (IC) in the dorsal midbrain is a key subcortical auditory structure (Aitkin and Phillips, 1984; Moore, 1987). Evidence from animal models suggests that IC is not only a major computational hub for ascending auditory inputs—perhaps comparable to V1 in the visual system (Nelken et al., 2003; King and Nelken, 2009)—but also a recipient of top-down signals from auditory cortex and thalamus as well as other brain regions implicated in multisensory and cognitive processing (Casseday et al., 2002; Gruters and Groh, 2012).

In animal models, the varying functional roles of IC are largely delineated by major anatomical subdivisions within IC. The central nucleus of the IC (ICc) receives the majority of ascending auditory inputs from the brainstem auditory structures, which arrive *via* the lateral lemniscus (Aitkin and Phillips, 1984; Moore, 1987; Winer et al., 1998; Markovitz et al., 2013; Ono and Ito, 2015). This lemniscal pathway contrasts with non-lemniscal pathways, which largely pass through the dorsal (ICd) and external (ICx) subdivisions of IC. These structures largely receive non-primary ascending auditory inputs as well as lateral and top-down inputs from other brain regions (Suga et al., 1997; Winer et al., 1998; Gruters and Groh, 2012; Straka et al., 2015; Carbajal and Malmierca, 2018; Ito and Malmierca, 2018; Suga, 2020). However, despite their unique functional roles, the major IC subdivisions share wide-ranging connections with both auditory and non-auditory structures (Aitkin, 1989; Oliver, 2005; Winer, 2005; Cant and Oliver, 2018).

While the animal literature on IC subdivision function and connectivity has been built over decades, research in humans has been limited due to the technical challenges of imaging small structures deep within the living human brain. Previous work has demonstrated the feasibility of functional localization and structural connectivity of human subcortical auditory structures *in vivo* (Devlin et al., 2006; Sitek et al., 2019), as well as fundamental sound response properties (Hawley et al., 2005; Sigalovsky and Melcher, 2006; De Martino et al., 2013; Ress and Chandrasekaran, 2013; Moerel et al., 2015). Recent work has investigated functional subdivisions of human auditory thalamus (Mihai et al., 2019; Tabas et al., 2020). However, to our knowledge no work has investigated subdivisions of human IC in living humans or their patterns of connectivity beyond IC.

Due to the lack of clarity in the literature regarding human IC subdivision connectivity, we sought to identify the white matter connectivity of human IC subdivisions using three high quality datasets to establish the feasibility of human IC subdivision connectivity measurements with diffusion-weighted MRI tractography. Using a 200 μm isotropic resolution *post mortem* sample, a high resolution 760 μm isotropic diffusion MRI (dMRI) dataset from a single living human participant, and a near-millimeter resolution 10-subject 7T dMRI dataset, we used *k*-means clustering to identify unique connectivity patterns of human IC. We then identified the major subdivisions of IC and estimated tractography from each subdivision. Our findings suggest that dMRI tractography is sensitive to fine-grained structural connectivity within human IC.

## Materials and Methods

### MRI Data Acquisition

Three unique datasets were included in this study. The first is a *post mortem* human brainstem of a 65-year-old male who died of non-neurological natural causes (Calabrese et al., 2015; Sitek et al., 2019; Rushmore et al., 2020; Adil et al., 2021). The tissue was removed approximately 24 h *post mortem* and fixed with 10% formalin solution for 2 weeks. The tissue was rehydrated in saline with 1% gadoteridol 1 week before MRI acquisition. For imaging purposes, the tissue was placed in a fluorocarbon liquid in a custom MRI-compatible tube. DMRI was collected in a small-bore 7-Tesla MRI over 208 h at *b* = 4,000 s/mm^2^ in 120 diffusion directions at 200 μm isotropic resolution. Anatomical T2*-weighted images were collected at 50 μm isotropic resolution.

The second dataset is a single *in vivo* participant (about 30 years old) scanned in a 3-Tesla Siemens Connectom scanner over 18 h at *b* = 1,000 and 2,500 s/mm^2^ in 1,260 diffusion directions at 760 μm isotropic resolution (see Wang et al., 2021 for complete acquisition details). The participant gave written informed consent for participation in the study, which was approved by the Institutional Review Board of Partners Healthcare.

Finally, we used an existing *in vivo* dataset of 10 individuals (25–30 years of age) scanned in a 7-Tesla Siemens Magnetom MRI at 1.05 mm isotropic resolution (Sitek et al., 2019). The dMRI acquisition was based on the 7T Human Connectome Project acquisition (Vu et al., 2015) and extended from two shells to three (*b* = 1,000, 2,000, 3,000 s/mm^2^) (Gulban et al., 2018). This experiment was approved by the ethics committee of Maastricht University (protocol number ERCPN-167_09_05_2016), and each participant provided written informed consent for participation in the study.

### Diffusion MRI Processing

Each volume of the *post mortem* dMRI data was affine-transformed to the first b0 image volume using ANTs tools (Avants et al., 2011) in order to correct for eddy current distortions (Calabrese et al., 2015). The *post mortem* dMRI data were then linearly transformed to the T2*-weighted anatomical MRI (Sitek et al., 2019).

The sub-millimeter *in vivo* images were corrected for susceptibility-induced distortion, eddy-current distortion, gradient non-linearity, and subject motion using FSL tools (Jenkinson et al., 2012) as described in Wang et al. (2021).

Diffusion orientation estimation for the *post mortem* and sub-millimeter *in vivo* MRI was performed in DSI Studio using generalized q-sampling imaging (GQI) (Yeh et al., 2010), including the generation of quantitative anisotropy maps [similar to fractional anisotropy (FA) maps generated in diffusion tensor imaging (DTI) analysis].

*Post mortem* dataset processing was performed locally on an Intel-based Macbook Pro using the 7 January 2021 build of DSI Studio; the *in vivo* sub-millimeter dataset was processed in DSI Studio by F. C. Yeh and shared publicly at https://brain.labsolver.org/mgh_760.html.

The 7T *in vivo* dataset was processed using the HCP pipeline (Sotiropoulos et al., 2013; Glasser et al., 2016), including geometric and eddy-current distortion correction and motion compensation. Data were masked to only include the brainstem and thalamus, with diffusion fiber orientation distributions estimated using constrained spherical convolution implemented in DIPY (Tournier et al., 2004; Garyfallidis et al., 2014). Streamlines were generated using DIPY’s EuDX approach.

### Data Analysis

We conducted two analyses in each dataset. In the unsupervised *k*-means clustering analysis, the entire IC was used as a tractography seed in DSI Studio (Yeh et al., 2010). The resulting streamlines were partitioned using *k*-means clustering, run with multiple values of *k* ranging from 2 to 10. In the seed-based analysis, IC subdivisions were manually labeled on the anatomical images, and tractography was run from each subdivision.

In both analyses, the anisotropy threshold was randomly selected between 0.5 and 0.7 times the Otsu threshold (maximizing contrast between foreground and background), the angular threshold was 90°, and the step size was randomly selected from 0.5 to 1.5 voxels. Each individual tractography operation used 10 million seed points with subvoxel seeding and utilizing all fiber orientations in each voxel.

### Segmenting Inferior Colliculus Subdivisions

We segmented IC following human histological literature utilizing a variety of staining techniques, including Nissl, myelin, and AChE (Moore, 1987; Webster, 1992; Mansour et al., 2019; Paxinos et al., 2020). Although there are slight differences in terminology throughout the literature, we divided IC into three major subdivisions: central nucleus of the inferior colliculus (ICc), external cortex of the IC (ICx), and dorsal cortex of the inferior colliculus (ICd).

**FIGURE 1:**
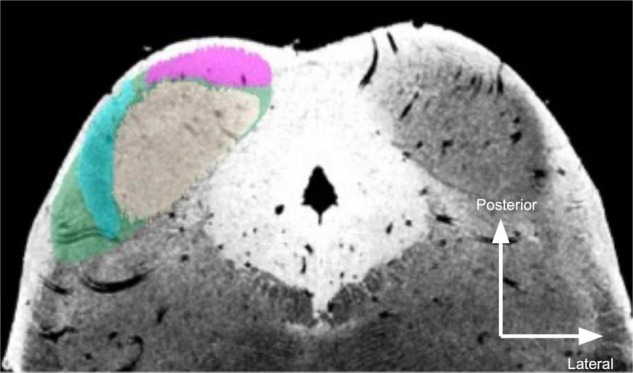
Segmentation of *post mortem* inferior colliculus and its major subdivisions. Neutral gray, central nucleus (ICc); magenta, dorsal nucleus (ICd); turquoise, external nucleus (ICx); green, overall inferior colliculus (IC). The right IC is unsegmented to show MR contrast. Segmentations were performed manually on the 50 μm isotropic T2*-weighted anatomical dataset and transformed to dMRI space (200 μm isotropic).

In the *post mortem* sample, overall IC segmentations were originally generated in an atlas of human subcortical structures (Sitek et al., 2019) and are publicly available at https://osf.io/c4m82/. For this investigation, we used the conjunction atlas (based on two raters) that was then dilated 500 μm (about 2.5 voxels)—see section “Discussion” for further discussion. Within IC, ICc was identified as the large, moderate intensity (in the T2*-weighted image) area near the center of each IC (Figure 1). ICx appeared as a dark band lateral to ICc in the T2*-weighted images. ICd was segmented as the hyperintense zone rostral to ICc.

**FIGURE 2:**
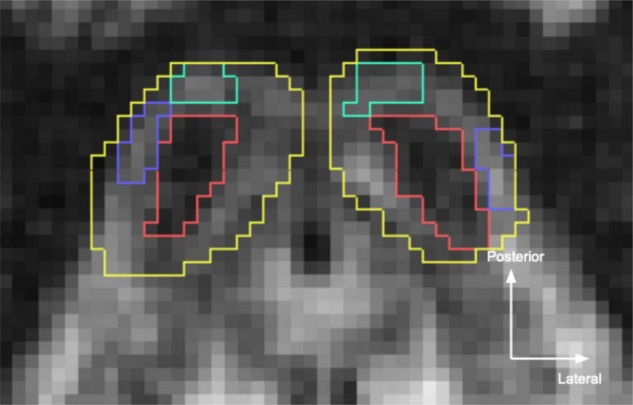
Inferior colliculus (IC) segmentations in the sub-millimeter *in vivo* dataset. Yellow, overall IC; red, central nucleus (ICc); turquoise, dorsal nucleus (ICd); blue, external nucleus (ICx). Segmentations were hand drawn on the 760 μm isotropic diffusion MRI quantitative anisotropy map.

In the *in vivo* datasets, IC was identified on axial views of the dMRI quantitative anisotropy map as caudal structures of the dorsal tectum, bounded by PAG ventrally, superior colliculus rostrally, and CSF dorsally and laterally (Figure 2). Within IC, ICc was visible on the quantitative anisotropy images a dark structure at the center of IC. Meanwhile, ICx could be found as a lighter band lateral to ICc, while ICd was identifiable as a lighter band posterior to ICc.

In dMRI tractography, streamlines (sometimes referred to as “tracks” or “tracts”) are volumeless representations of likely white matter pathways. To determine the paths of streamlines beyond IC, we segmented three white matter structures adjacent to IC: lateral lemniscus, which connects IC with the more caudal brainstem auditory structures; brachium of the IC, which connects IC with more rostral thalamic and cortical auditory structures; and commissure of the IC, which connects the two colliculi. To compare connectivity patterns between IC subdivisions, we then counted the number of streamlines from each IC subdivision to each white matter structure.

To assess the specificity of *k*-means clustering streamline clusters with respect to anatomically defined IC subdivisions, in the 7T *in vivo* dataset, we assessed the number of streamlines per *k*-means cluster (with the number of clusters ranging from *k* = 2–10) that passed through each IC subdivision. For each IC, *k*, and cluster with sufficient streamlines, we computed an ROI–streamline FA score, using the number of streamlines passing through each IC subdivision. This provides a quantitative value (from 0 to 1) that represents the a given streamline cluster’s specificity for each IC subdivision and allows us to discern whether a particular number of *k*-means clusters is optimally aligned with anatomically segregated streamlines.

However, there is debate about the utility of streamline counts, which vary based on acquisition and analysis details (Smith et al., 2012, 2015, 2020; Yeh et al., 2019; Rheault et al., 2020); specific values should be interpreted with caution.

## Results

### Diffusion MRI Tractography of Inferior Colliculus

**FIGURE 3:**
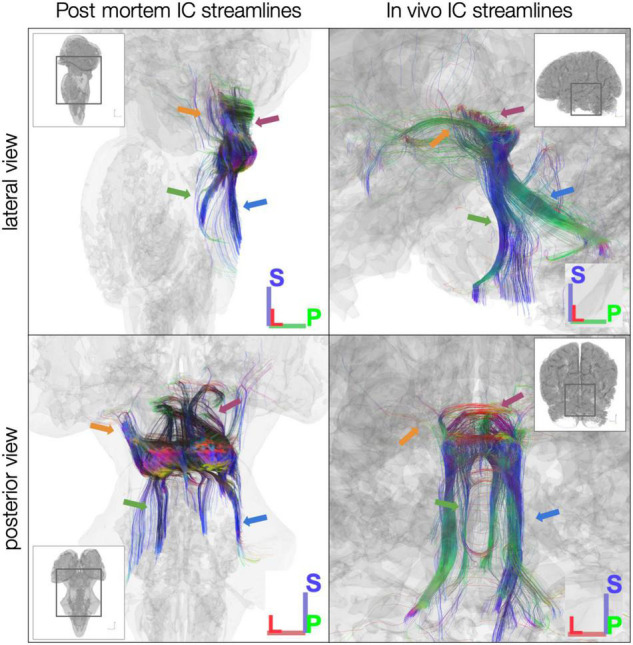
Diffusion MRI tractography streamlines passing through left and right inferior colliculus (IC). Colors represent local streamline orientation: blue, superior–inferior; red, left–right; green, anterior–posterior. Insets show entire specimen or brain surface. Left: *post mortem* dataset. Right: *in vivo* MGH 760 μm dataset. Both datasets demonstrate canonical auditory pathways (green arrows, lateral lemniscus; orange arrows, brachium of IC) as well as non-primary auditory connections [for instance, to superior colliculus (magenta arrows) and through cerebellar peduncles (blue arrows)].

We first ran tractography using the entire IC as a seed ROI. Starting with 10 million seed points, in the *post mortem* dataset, we generated 3,707 streamlines from left IC and 5,143 streamlines from right IC (Figure 3, left). In the sub-millimeter *in vivo* dataset, we generated 629 streamlines from left IC and 1,017 streamlines from right IC (Figure 3, right). In the 7T *in vivo* dataset (which was preprocessed separately and modeled using constrained spherical deconvolution as opposed to generalized GQI like the *post mortem* and sub-millimeter *in vivo* datasets), averaged across the 10 participants, we generated 95.4 left IC streamlines and 98.4 right IC streamlines. Overall, from visual inspection of the generated dMRI results (Figure 3), streamlines that pass through IC run caudally through lateral lemniscus to the brainstem auditory structures, rostrally through the brachium of the IC to the thalamic (medial geniculate) and cortical auditory structures, and laterally through the commissure of the IC to the contralateral IC. In addition, we identified streamlines passing through non-primary auditory structures, such as superior colliculus (rostral/superior relative to IC) and cerebellar peduncles (caudal/inferior and posterior relative to IC; Figure 3).

### *k*-Means Clustering of Inferior Colliculus Streamlines

**FIGURE 4:**
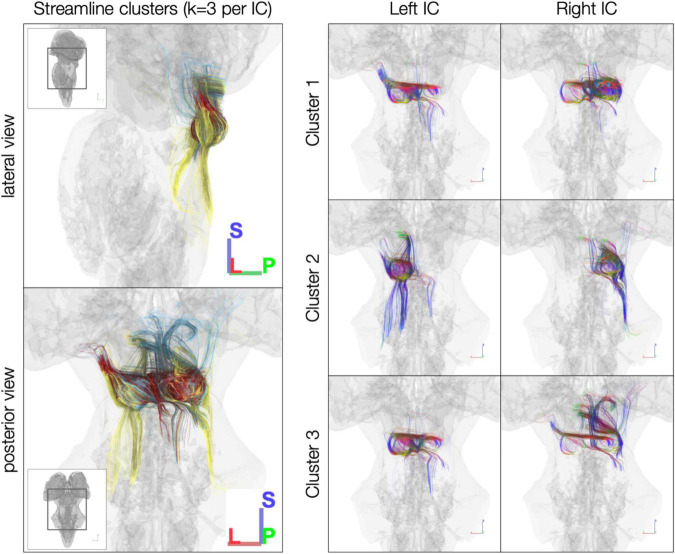
*k*-Means clustering of inferior colliculus (IC) streamlines (*k* = 3 per IC). Left: color denotes cluster (1, 2, or 3, based on *k*-means clustering). Right: color denotes local streamline orientation: blue, superior–inferior; red, left–right; green, anterior–posterior.

In the *post mortem* dataset, *k*-means clustering isolated streamlines rostrally between IC and thalamus/cortex and caudally between IC and brainstem (Figure 4). Although clustering was performed separately for each IC, the three resulting clusters were similar across left and right ICs. For instance, Cluster 2 in each IC consisted largely of caudal streamlines, with limited rostral streamlines and very few commissural streamlines (Figure 4, right). Meanwhile, Clusters 1 and 3 exhibited more commissural streamlines in both ICs.

**FIGURE 5:**
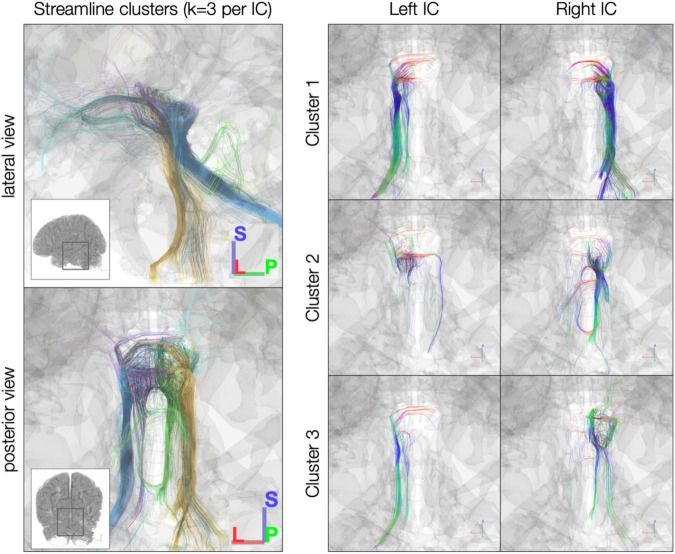
*k*-Means clustering [*k* = 3 per inferior colliculus (IC)] of the *in vivo* 760 μm dataset. Left: color denotes cluster (1, 2, or 3, based on *k*-means clustering). Right, color denotes local streamline orientation; blue, superior–inferior; red, left–right; green, anterior–posterior.

In the sub-millimeter *in vivo* dataset, the reduced number of streamlines limit the interpretability of the *k*-means clustering results, although the clusters in left and right IC have similar connectivity patterns despite being generated in separate *k*-means clustering operations (Figure 5). In left and right IC, Cluster 1 contains primarily caudal-extending streamlines that also reach toward the midline at the level of the IC or SC. Cluster 2 in both ICs captured many of the cerebellar streamlines, while Cluster 3 has predominantly rostral-extending streamlines that also extend caudally toward brainstem and cerebellum.

### Anatomically Defined Inferior Colliculus Subdivision Connectivity

**FIGURE 6:**
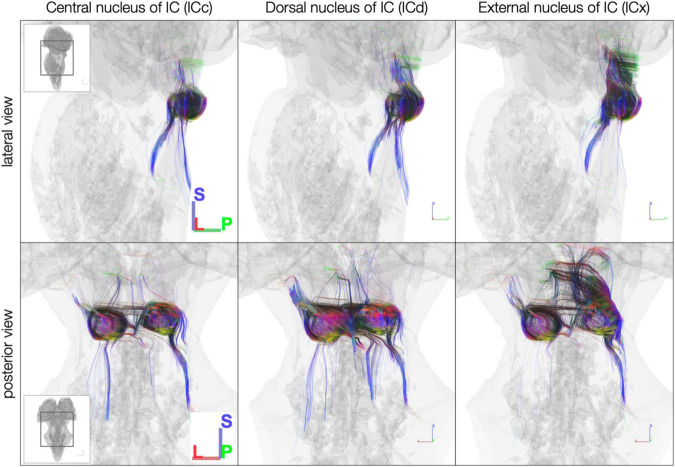
Diffusion MRI tractography streamlines reaching each of the inferior colliculus subdivisions in the *post mortem* dataset. Colors represent local streamline orientation: blue, superior–inferior; red, left–right; green, anterior–posterior.

Using anatomically defined IC subdivisions as tractography seeds, streamlines were less extensive than when using the whole IC segmentations as tractography seeds. In the *post mortem* dataset, the central nucleus of the IC (ICc) had the fewest streamlines extending rostrally toward the medial geniculate body (MGB) of the thalamus (Figure 6). Meanwhile, the dorsal nucleus of the IC (ICd) had by far the most streamlines crossing the midline to the contralateral IC. Additionally, the external nucleus of the IC (ICx) exhibited more streamlines extending rostrally toward MGB than caudally toward brainstem.

**FIGURE 7:**
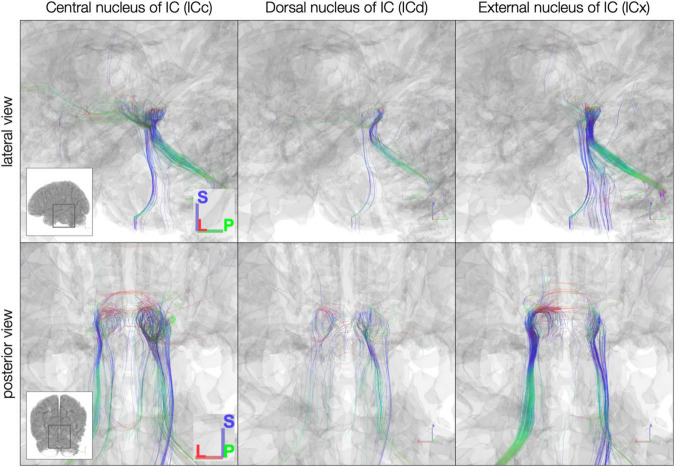
*In vivo* anatomically defined inferior colliculus (IC) subdivision streamlines. Colors represent local streamline orientation: blue, superior–inferior; red, left–right; green, anterior–posterior.

The sub-millimeter *in vivo* dataset (Figure 7) demonstrated sparser connectivity than the *post mortem* dataset to IC subdivisions. Unlike the *post mortem* dataset, few streamlines from any subdivision extended rostrally toward thalamus and cortex or crossed the midline to the contralateral IC. Caudal-extending streamlines were more frequent, particularly from ICc and ICx on both the left and right.

**FIGURE 8:**
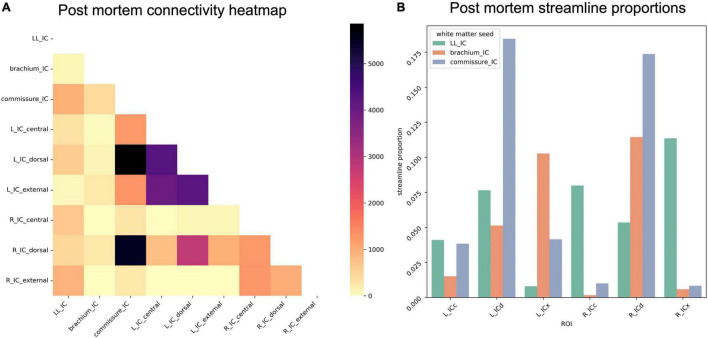
**(A)** Connectivity matrix of *post mortem* inferior colliculus (IC) subdivisions and adjacent white matter tracts (values indicate number of streamlines); **(B)** proportion of streamlines reaching each white matter region for each IC subdivision.

To assess overall connectivity patterns, we counted the number of streamlines from each IC subdivision that reached each white matter structure (lateral lemniscus, brachium of the IC, and commissure of the IC). Of the white matter structures, only the IC commissure showed a preference for streamlines from any particular IC substructure in the *post mortem* dataset, with a large proportion of commissural streamlines passing through ICd contralaterally (Figure 8).

**FIGURE 9:**
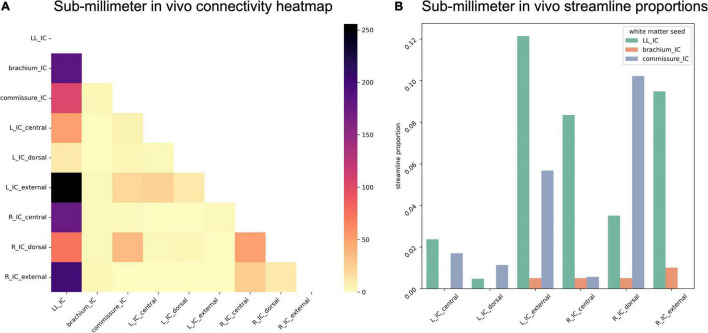
**(A)** Connectivity matrix of sub-millimeter *in vivo* inferior colliculus (IC) subdivisions and adjacent white matter tracts (values indicate number of streamlines); **(B)** proportion of streamlines reaching each white matter region for each IC subdivision.

Due to the reduced number of overall IC streamlines in the sub-millimeter *in vivo* dataset, we found fewer streamlines from IC subdivisions reaching the white matter structures (Figure 9). Right ICd contributed the most streamlines to commissural connections, but interestingly not left ICd. Compared to the *post mortem* dataset, a higher proportion of lateral lemniscal streamlines passed through IC substructures, particularly left and right ICx (as well as right ICc).

**FIGURE 10:**
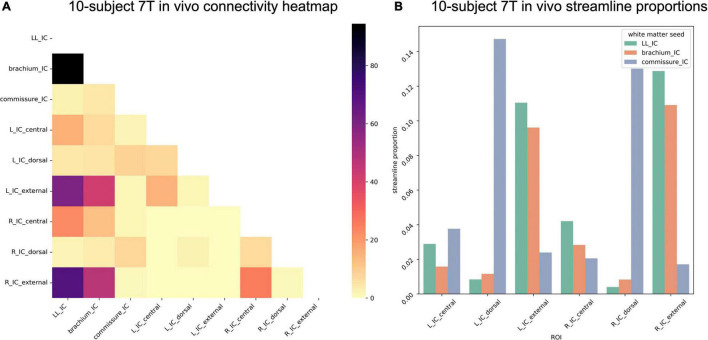
**(A)** Connectivity matrix of 7T *in vivo* inferior colliculus (IC) subdivisions and adjacent white matter tracts (values indicate number of streamlines averaged across 10 participants); **(B)** proportion of streamlines reaching each white matter region for each IC subdivision.

In the 10-participant 7T *in vivo* dataset, we again saw strong commissural connections through dorsal IC (Figure 10). In this dataset, the brachium of the IC and the lateral lemniscus shared connectivity patterns, with similar streamline proportions reaching central and external IC nuclei.

### Relating *k*-Means and Anatomical Approaches

To assess whether a particular number of *k*-means clusters is optimal for segregating subdivision streamlines, we ran *k*-means clustering with *k* varying from 2 to 10 on streamlines passing through each IC in the 10-subject *in vivo* dataset. We counted the number of streamlines in each cluster passing through each anatomically defined IC subdivision and, to determine whether a given cluster had a specific subdivisional correspondence, calculated a “ROI–subdivision fractional anisotropy” score for each *k*-value and IC. Upon inspection, many of the clusters at higher *k*-values had few or no streamlines, due to the small number of streamlines to begin with. We therefore focused our investigation on *k* = 2–5. Across *k*-values from 2 to 5, the mean score across subjects ranged from 0.61 to 0.69. We did not find a significant difference between ROI–subdivision FA scores from different *k*-values (Kruskal–Wallis *h* = 0.50, *p* = 0.92), suggesting there was not an optimal number of clusters that aligned best with subdivision anatomy.

## Discussion

Using sub-millimeter and near-millimeter resolution dMRI from high quality *post mortem* and *in vivo* human datasets, we investigated substructure connectivity patterns of human IC. As gold standard methods such as tracer injections are challenging or impossible with human tissue, dMRI tractography represents the best opportunity to map fine-grained connections in the human brain (Barbeau et al., 2020). With unsupervised *k*-means clustering approaches to cluster white matter connections through IC, we found that streamline clusters were segregated based on their origin beyond IC as well as their location within IC. For instance, in both the *post mortem* and *in vivo* datasets, caudal-extending streamlines (toward brainstem through the lateral lemniscus) were largely separated into their own cluster, reaching the IC in distinct locations. The results aligned with connectivity patterns based on tractography analysis using anatomically defined IC subdivisions: both *post mortem* and *in vivo* datasets demonstrated lateral lemniscal streamlines through central and external IC nuclei, as well as strong commissural connections through dorsal IC nuclei. Taken together, the *k*-means clustering and anatomically driven analyses demonstrate that dMRI tractography can reveal fine-grained structural connectivity patterns within the human subcortical auditory system. By utilizing diverse state-of-the-art datasets with results largely in agreement, our results help build consensus around the utility of dMRI tractography for investigating subcortical auditory connectivity.

Although many of the streamlines we observed in tractography results align with the major auditory pathways (including lateral lemniscus and brachium of the IC), we also identified streamlines heading toward non-auditory structures (Figure 3). For instance, in both our *post mortem* and *in vivo* datasets, superior colliculus received many streamlines that were generated with a tractography seed place in the external nucleus of IC (ICx), whose connections to superior colliculus have been described previously in animal models (Edwards et al., 1979; Druga and Syka, 1984; Doubell et al., 2000; Bednárová et al., 2018). Similarly, both datasets showed IC streamlines running toward cerebellum (Powell and Hatton, 1969). As we continue mapping and quantifying the structural connectivity of human IC, it is important to keep in mind that auditory processing plays a critical role in motor, limbic, and multisensory processing, and the connections with non-primary auditory structures that have been previously identified in animal models may be crucial infrastructure for these complex neural functions.

This work builds on previous literature examining the structural connectivity of human IC as a whole. Some groups have used probabilistic tractography to estimate connectivity between other auditory structures and IC (Devlin et al., 2006; Javad et al., 2014). Others used IC as a landmark for estimating subcortical auditory connectivity in clinical populations (Lin et al., 2008; Wu et al., 2009; Tarabichi et al., 2018). Our own previous work established reliable *post mortem* and *in vivo* tractography estimates of connectivity throughout the subcortical auditory system, including to IC (Sitek et al., 2019). However, to our knowledge, no previous work has investigated the connectivity patterns of IC’s constituent nuclei.

Much of this difficulty arises from accurately segmenting the anatomical boundaries of IC subdivisions in living humans. Indeed, we did not find any previous MRI investigations of *in vivo* human IC subdivisions, and *post mortem* MRI atlases have been varying in detailing IC substructure (Paxinos et al., 2012). In the present work, we take advantage of the high MR contrast and ultra-high resolution *post mortem* MRI to finely delineate the major subdivisions of human IC, which we then used as the basis for segmenting the IC subdivisions in the included *in vivo* dataset.

Despite the advances of the present work, applying the methods to standard *in vivo* diffusion-weighted MRI pose significant challenges. Each of the datasets used in this study was acquired over multiple days in unique MRI environments with specialized scanning protocols. In contrast, diffusion-weighted MRI is typically collected in a single session—often in just one or two 5–10-min acquisitions—on standard 3T MRI scanners, which limits the potential spatial resolution, angular resolution, diffusion sensitivity, and contrast-to-noise ratio of the collected images (McNab et al., 2013). Further, there remain outstanding issues in the implementation and interpretation of dMRI tractography, such as a lack of specificity resulting in many false positives (Thomas et al., 2014; Schilling et al., 2019). Additionally, there are unresolved issues when quantifying streamlines and connections (Jbabdi and Johansen-Berg, 2011; Jeurissen et al., 2019; Smith et al., 2020), which limits the interpretation of specific streamline counts in the present study. However, advances in dMRI acquisition and analysis, including at ultra-high magnetic fields, are improving the sensitivity and reliability of dMRI tractography (Setsompop et al., 2013; Sotiropoulos et al., 2013; Vu et al., 2015; Jeurissen et al., 2019; Kruper et al., 2021; Moeller et al., 2021; Yeh et al., 2021), making finer-grained connectivity investigations more accessible to the broader neuroimaging community. Additional *post mortem* human dMRI datasets (Edlow et al., 2019; Tendler et al., 2021)—along with complementary cellular-level resolution methods such as polarized light imaging (Axer et al., 2011) and polarization-sensitive optical coherence tomography (Jones et al., 2020)—will provide critical details on human brainstem 3-D anatomy.

In general, sub-millimeter resolution dMRI is necessary for brainstem tractography in order to dissociate densely packed nuclei and white matter pathways (Ford et al., 2013; Grisot et al., 2021; Yendiki et al., 2021). However, imaging at high resolution may introduce its own challenges in identifying connectivity between nuclei, particularly in brainstem and other non-cortical brain structures. For example, as we have previously discussed (Sitek et al., 2019), the improved contrast and spatial specificity between gray matter and white matter results in decreased partial volume effects, with a (unsurprising, but perhaps overlooked) result of streamlines staying within the white matter and fewer streamlines reaching finely segmented gray matter structures. This may have affected our present results, where the anatomically defined subdivision segmentations generally excluded adjacent white matter and thus may not demonstrate the full connectivity patterns of these regions. For this reason, we opted to use the 500 μm-dilated whole IC segmentation from Sitek et al. (2019), which demonstrated improved connectivity profiles relative to the strict IC gray matter segmentation.

However, with the development of MRI hardware capable of stronger diffusion encoding, as well as continued research into optimal preprocessing and analysis methods, we are hopeful that IC subdivision tractography will yield new insights into the relationship between subcortical auditory connectivity and perception-dependent human behavior such as speech communication and music, as well as the role of human IC subdivisions in health and disease.

## Data Availability Statement

The datasets presented in this study can be found in online repositories. The names of the repository/repositories and accession number(s) can be found below: https://github.com/SoundBrainLab/IC_subdivision_connectivity.

## Ethics Statement

The studies involving human participants were reviewed and approved by the Institutional Review Board of Partners Healthcare and the Ethics Committee of the Faculty of Psychology and Neuroscience of the University of Maastricht. The patients/participants provided their written informed consent to participate in this study. Written informed consent was obtained from the individual(s) for the publication of any potentially identifiable images or data included in this article.

## Author Contributions

KS analyzed the data, wrote the manuscript, and submitted the manuscript. EC and GJ collected and preprocessed the *post mortem* data and provided feedback on the manuscript. SG provided guidance on analysis and feedback on the manuscript. BC provided guidance on analysis, contributed to and provided feedback on the manuscript, and provided funding for the research. All authors contributed to the article and approved the submitted version.

## Conflict of Interest

The authors declare that the research was conducted in the absence of any commercial or financial relationships that could be construed as a potential conflict of interest.

## Publisher’s Note

All claims expressed in this article are solely those of the authors and do not necessarily represent those of their affiliated organizations, or those of the publisher, the editors and the reviewers. Any product that may be evaluated in this article, or claim that may be made by its manufacturer, is not guaranteed or endorsed by the publisher.
